# Structural Characteristics of the Iranians Nose: An Anatomical Analysis

**DOI:** 10.22038/ijorl.2020.42574.2391

**Published:** 2020-09

**Authors:** Jahangir Ghorbani, Ali Safavi Naeini, Golfam Mehrparvar, Atsa Doroudinia, Shahram Kahkouee, Habib Emami

**Affiliations:** 1 *Chronic Respiratory Diseases Research Center, Masih Daneshvari Hospital, Shahid Beheshti University of Medical Sciences, Tehran, Iran.*; 2 *Department of Otolaryngology Head and Neck Surgery, Masih Daneshvari Hospital, Faculty of Medicine, Shahid Beheshti University of Medical Sciences, Tehran, Iran.*; 3 *Department of Pathology, Faculty of Medicine, Shahid Beheshti University of Medical Sciences, Tehran, Iran.*; 4 *Department of Radiology, Faculty of Medicine, Shahid Beheshti University of Medical Sciences, Tehran, Iran.*; 5 *Department of Epidemiology, Faculty of Medicine, Shahid Beheshti University of Medical Sciences, Tehran, Iran.*

**Keywords:** Anatomy, Cartilage, Subcutaneous Fat, Rhinoplasty

## Abstract

**Introduction:**

Rhinoplasty is one of the most common surgical procedures performed among Iranians. An important issue to be considered by nasal surgeons is anatomical variations between different ethnic groups. Working on Iranians with the existing ethnic variety encourages the need for an analysis of this particular population.

**Materials and Methods:**

The present cross-sectional observational study was conducted on Iranian patients who underwent primary open rhinoplasty at a university hospital in Tehran, Iran. The preoperative evaluations included routine aesthetic analysis as well as the measurement of the subcutaneous fat thickness (using ultrasound imaging) and the angle between the anterior nasal spine and the alveolar process of the maxilla. Intraoperative assessments were performed on the alar rim-inferior border of lateral crus distance, maximal width of lateral crus, connection pattern of upper lateral cartilage, and lower lateral cartilage. Alar cartilage thickness was measured with microscopic evaluation. Moreover, the similar studies conducted on other ethnic groups were reviewed as well.

**Results:**

In total, 66 cases were included in the study (41 females and 25 males) who were within the age range of 18-38 years old (27.82±5.51). According to the results, nasolabial angles were 93.68°±7.82° and 92.25°±6.98° in females and males, respectively. In addition, a significant correlation was found between the anterior nasal spine-maxilla angle and nasolabial angle (P<0.05). Moreover, the findings revealed a significant but weak negative correlation between alar subcutaneous fat thickness and alar cartilage thickness (0.0002). Maximal width of lateral crus was found to be 11.44mm±2.02 and 10.41 mm±1.72 in males and females, respectively.

**Conclusion:**

Despite the differences observed between various ethnic groups, drawing a definiteconclusion about these variations needs comparative studies with similar samples (cadaver vs. patients) and measurement techniques.

## Introduction

Rhinoplasty is one of the most challenging plastic surgical procedures and is predominantly performed to improve nasal appearance. Therefore, the goal of preoperative planning is to make changes in the bony and cartilaginous framework to create the desired morphology. However, the final appearance will be the result of not only the shape of underlying structures but also the characteristics of soft tissue envelope and the interactions between the two. Another issue that is of great importance is the changes in the postoperative period which sometimes show an unpredictable course which makes it difficult to estimate the final result.

Undoubtedly, a comprehensive understanding of surgical anatomy is the primary step towards a perfect procedure. Despite the fact that the anatomy seems static, the continual innovation of new techniques and approaches makes this field very dynamic (1). An important issue to be considered in facial plastic surgeries, including rhinoplasty, is the anatomical differences between different ethnic groups, which require distinct procedures. However, it could be deleterious to follow the surgical principles mentioned in references that are based on observations in special ethnic groups and generalize them. Despite similarities in general structural characteristics, different ethnic groups have significant differences regarding their anatomical details. There are some broad classifications in plastic surgery literature according to these anatomical variations (e.g., Caucasians vs. non-Caucasians). However, some ethnic groups cannot be precisely included in any one of these two categories. 

Rhinoplasty is one of the most common plastic surgical procedures performed among Iranians. Performance of such surgeries on Iranians with the existing ethnic variety encourages the need for an analysis of this particular population.

## Materials and Methods

This study was conducted at a university medical center in Tehran, Iran on Iranian cases who underwent primary open rhinoplasty over six months (September 2018 to March 2019). The study was approved by the ethics committee of the national research institute of Masih Daneshvari Hospital, Shahid Beheshti University of medical sciences, Tehran, Iran.

The exclusion criteria consisted of 1) history of previous nasal surgery or significant nasal trauma, 2) congenital facial deformities, 3) evidence of nasal skin /soft tissues scar, 4) underlying disorders affecting the skin, soft tissues, and bony and cartilaginous structures, 5) non-Iranian ancestors (based on history), 6) and 7) lack of consent to participate in the research. For the purposes of the study, routine preoperative workups were performed, including analysis of standard photographic views. 

Moreover, nasolabial angles were specifically measured in the lateral (profile) view. The subcutaneous fat thickness on alar and tip area was measured via sonography by a single radiologist using a 12 MHZ linear probe (Mind ray Bio-Medical Electronics Co, Ltd, Shen Zhen, China).

Ultrasound evaluation was performed following the application of a topical gel, without inserting pressure on the probe (to prevent soft tissue distortion). Subcutaneous fat thickness was measured on left and right alar areas, and then the average of the two was calculated in each case. Similarly, the subcutaneous fat thickness on the tip area was measured from the most prominent point on the dome area to the skin layer. 

In cases with available cross-sectional imaging (computed tomography), the scout view was evaluated in order to measure the angle between the anterior nasal spine and the anterior surface of the maxilla. The angle was defined as the intersection of a line along the alveolar process of maxilla with a line drawn from the anterior nasal spine. 

All of the patients underwent open rhinoplasty. Dissection was performed through transcolumellar incision, followed by elevation of skin soft tissue envelope. Subsequently, intraoperative photographs were taken. The following measurements were recorded after skeletonization:

- The distance from alar rim to the inferior border of lateral crus in 3 points (medial, middle and lateral)
- The maximum width of the lateral crus
- The patterns of connection between upper lateral cartilage and lateral crus which were categorized as follows:

No connection, end to end connection, overlap, scrolling (the caudal edge of upper lateral cartilage is scrolled under the surface of lateral crus), reverse scroll (the caudal edge of upper lateral cartilage is scrolled over lateral crus).

The alar cartilage thickness was measured by microscopic evaluation of the cephalic portion of lateral crus if resected and was not required as a graft for other parts of the operation. This cartilaginous part was sent in formalin solution to the histopathology department and after preparation and staining, it was examined by a pathologist. 

The maximum cartilage thickness was reported in millimeters for each sample. The statistical analysis was performed in SPSS software (version 21) using the student’s t-test for the comparison of mean values. Moreover, the correlation of quantitative parameters (subcutaneous fat thickness of alar area with alar cartilage thickness, and nasolabial angle with the anterior nasal spine-maxillary angle) was investigated using Pearson correlation coefficient. 

## Results

A total number of 66 cases were included in the study consisting of 41 females and 25 males. The participants were within the age range of 18-38 years old (27.82±5.51). Furthermore, the mean and standard deviation of nasolabial angles were 93.68±7.82 and 92.52±6.98 in females and males, respectively. No statistically significant difference was observed between males and females (P=0.53) 

Sinonasal computed tomography (CT) was available from previous workups in only 49 patients (31 females and 18 males). 

Table 1 shows the values of anterior nasal spine-maxilla angle and their comparison with the nasolabial angle which demonstrates a significant correlation.

 Cephalic portion of the lateral crus was used as a graft in five cases and could not be evaluated. The microscopic measurement of alar cartilage thickness was performed on 38 females and 23 males. A statistically negative correlation was found between the alar subcutaneous fat thickness and alar cartilage thickness (Table.2). The connection patterns of upper lateral cartilage and lower lateral cartilage are summarized in table 3. 

Maximum width of lateral crus was found to be 11.44mm±2.02 and 10.41mm±1.72 in males and females, respectively. Table 4 demonstrates the obtained data about the distance between the caudal edge of alar cartilage to nostril border in medial, middle, and lateral parts based on gender.

**Table 1:** Correlation of nasolabial angle* with the angle between the anterior nasal spine and the anterior surface of maxilla**

**Variable **	**Female** **(n=31)**	**Male ** **(n=18)**
NLA^*^	92.52º	93.68º
ANS -P A^**^	83.56º	88.44º
R^***^	0.606	0.32
P Value	0.0013	0.043

**Table 2:** Correlation between subcutaneous fat thickness and alar cartilage thickness

**Variable **	**Total **	**Female (n=38)**	**Male (n=23)**
Alar subcutaneous fat thickness	2.48±0.49	2.32±0.49	2.37±0.51
Alar cartilage thickness	0.58±0.08	0.47±0.08	0.51±0.1
R			-0.0134
P-value			0.0002

**Table 3:** **:** ^* ^Relationship of upper lateral cartilage with lower lateral cartilage. D: disconnected, E: end to end, O: overlap, S: scroll, RS: reverse scroll

**Female(n=41)**		**Male (n=25)**	**Variable**
ULC-LLC*	D	2(8%)	9(22%)
	E	7(28%)	10(24.4%)
	O	10(40%)	13(31.7%)
	S	6(24%)	9(22%)
	RS	0	

**Table 4:** The distance from caudal border of lateral crus and alar rim

**Female(n=41)**	**Variable**	**Male (n=25)**	**P** ^*^
Medial	4.38±0.66	5.38±1.22	0.0001
Middle	6.93±1.16	7.9±1.44	0.003
Lateral	10.89±1.55	11.9±1.90	0.02

*P values for difference between males and females

## Discussion

The first essential principle of any surgery is the comprehensive anatomic knowledge of the selected area (1). Satisfactory rhinoplasty outcome to a great extent depends on the knowledge of the nasal anatomy, both bony-cartilaginous framework and soft tissue envelope. Assessment of different aspects of nasal anatomical structures has been considered by different researchers and related articles are evident in anatomy, plastic surgery, maxillofacial, and otorhinolaryngology literature. Recently the racial anatomical differences have attracted the interest of researchers which has resulted in various studies performed mostly on cadavers or imaging data. In this regard, Rohrich and Ghavami analyzed the Middle Eastern nose. They evaluated photographs and intraoperative findings in a 3-year retrospective study and considered patients from north African countries, gulf countries (including Iran), and others (Turkey, Lebonan, Syria, Armenia, Afghanistan, Pakistan, and India) as Middle Easterners. Their study revealed some characteristics of the Middle Eastern nose, such as thick skin, high dorsum, significant hump, ill-defined tip, weak lower lateral cartilages, and acute nasolabial angle (2). However, the ethnic subgroups categorized as belonging to Middle East countries may have individualized anatomic characteristics as well. Therefore, it would be more reasonable to describe them as separate groups.It is a common mistake to disregard the importance of the skin-soft tissue envelope that is observed through the analysis of unsatisfactory results. Skin-soft tissue thickness is a major factor contributing to the technique selection and surgical outcome. Soft-tissue thickness can be measured by several methods like radiography (3,4), caliper (5), and ultrasound imaging (6). Cho et al. believed that a CT scan with the ability to detect small attenuation differences could be a suitable method for the measurement of overlying soft tissue thickness. They evaluated 77 Korean cases and reported their mean nasal skin thickness to be 2.9mm for the nasal tip (7). 

Ultrasound evaluation of the subcutaneous soft tissue of nasal tip was first introduced by Tasman and Helrig in 2000 for the evaluation of post-surgical changes (8). They applied B-mode sonograph with a 12 megahertz transducer for this purpose. In their study, the average soft tissue thickness covering the surface of lower lateral cartilage was reported as 0.32 cm in 18 Caucasians. In comparison with our study, these Caucasian samples showed significantly thicker soft tissue over lateral cartilage (P<0.0001). However, Tasman did not mention the values for males and females separately and the sample size was too small to represent all Caucasians. 

The next studies showed the reliability and consistency of ultrasound imaging in the assessment of subcutaneous fat thickness (9, 10). The study of Naves and colleges in Brazil was aimed to determine the interobserver consistency of ultrasound evaluation (9). The authors did not mention the racial characteristics of participants who also had a wide age range (18-70 years). The results indicated an average of 4.3 mm subcutaneous soft tissue thickness on the tip area. It was significantly greater than the value of the subcutaneous fat thickness of the tip area in the present study (3.87±0.44) (P<0.0001).

Kosins et al. in a study on 75 Caucasian and non-Caucasian patients with the mean age of 33 years in California evaluated the soft tissue pocket. They used ultrasound imaging to measure the soft tissue thickness on the tip area in patients. The results of their study were categorized as thin (1.2 mm), moderate (2 mm), and thick (2.4 mm). Thick soft tissue pocket was more common among non-Caucasians (11). According to the review of the related literature, there are only a few studies performed on Iranian subjects. Nemati et al. conducted a case-control prospective clinical trial to evaluate the long-term results of nasal tip skin defatting and reported the nasal tip skin (not the subcutaneous fat) thickness of the cases as 3.2 (thick), 3.1 (moderate), and 2.6 mm (thin) (12). In the present study, the subcutaneous soft tissue thickness on the tip and alar area was measured by ultrasound imaging. The average thickness of the soft tissue of the tip area and the alar region were 3.87 and 2.37mm, respectively. We observed a statically significant difference between males and females with respect to subcutaneous soft tissue thickness on tip area (P=0.01), while the same was not true about those in the alar area.Upper and lower lateral cartilages need special attention as a part of the nasal valve area. Upper lateral cartilage is firmly connected to the nasal bone cephalically and is related to lateral crus of lower cartilage caudally. The degree of overlap between nasal bone and upper lateral cartilage should be kept in mind while reducing hump to prevent injury to the keystone area. Lower lateral cartilages almost always need modifications for tip plasty and their major role in the shape of the lower third and also nasal airway patency clarifies the importance of their morphology.

Connection patterns of upper and lower lateral cartilages were classified in a study by Yoo et al. on 30 Korean cadavers with an average age of 72 (13). They introduced five types of connections. The most common type in the aforementioned study was disconnection followed by overlap, scroll, and reverse scroll.

Kim in a similar study on 21 Korean cadavers and compared the results with whites (14). The results demonstrated that the length of upper and lower lateral cartilages in Koreans was similar to those in whites, while their widths were substantially smaller.

Connection patterns of upper lateral cartilage and lower lateral cartilage were also mentioned in their study. The most common pattern was interlocked (59.5%), followed by overlap (16.7%), end to end (11.9%), and not joined to each other (11.9%). Dion et al. (15) reported an 11% prevalence rate for reverse scroll pattern in their study on whites.Results of the present study revealed the following order of prevalence: scroll (34.8%), end to end (25.7%), overlap (22.7%), and disconnection (16.4%). The reverse scroll pattern was not found among the cases of the present study. In an anthropometric study of alar cartilage in Asians carried out by Dhong (16), the length, width, and thickness of lateral crus and the distance of lateral crus from medial nostril rim were compared in white and black people. The authors concluded that alar cartilages in Asians are not remarkably smaller than those of white people. However, there were differences regarding the configuration of the cartilage. The distance from the lateral crus to the alar rim was 5.8mm medially, 6.9mm centrally, and 11.8mm laterally. The same values were 6.5 and 13 mm in whites for medial and lateral measures respectively.A study carried out by Dhong (on Koreans) found that the lateral crural width were 10.5 and 11-12.8mm in whites (17,18), 12 mm in blacks (19), and 7-14 mm (mean:10mm) in Europeans (20). Based on the results of a study conducted by Daniel on Europeans, their lateral crural thickness and alar rim to alar cartilage in mid-nostril point were 0.5 and 3-9 mm, respectively (mean: 5.9 mm) According to the literature review of the Iranian studies performed on the cartilaginous morphology, there was a study performed by Farahvash et al (21) which dealt with the anatomical characteristics of lower lateral cartilage and compared it with other races. Based on the cadaveric dissections of 36 Iranian samples, they reported the lateral crural thickness was 1mm and the distance of lateral crus to nostril rim in medial, middle, and lateral points were 6, 7, and 14mm in males and 5, 6, and 12mm in females, respectively. 

Due to the estimated submillimeter thickness of the lateral crus, we decided to use microscopic evaluation with the hope of achieving more accurate results. The mean lateral crus thickness in our study was 0.51mm in all the samples collectively which showed a significant difference between males and females (P<0.0001). 

The mean value of the distance from the lateral crural border to nostril rim in the 3 medial, middle, and lateral points were 4.88, 7.29, and 11.39mm, respectively. These values should be kept in mind when doing the marginal incision to avoid soft tissue triangle injury. One must be cautious when comparing the results and the interpretation revealed by several studies available in the literature which deal with anatomical and morphological evaluations.In the studies performed on cadavers, we usually encounter old samples. 

On the other hand, since the cases are cadavers there are some changes in their soft tissue and cartilage characteristics which should be borne in mind when reviewing the results. Differences in the populations under study (cadavers-fresh vs. fixed-or patients), such as their mean age, gender, and ethnicity, as well as methods of measurement (intraoperative observations, microscopic or 

imaging-based measurements), are all important factors that need to be considered in the analysis of the data. Table 5 summarizes the results of similar studies on different ethnic groups. 

**Table 5:** Summary of results of similar studies performed on different ethnic groups

**STT**	**LCT** **(mm)**	**LCW** **(mm)**	**LC-nostril** **mm**			**Method**	**Gender Distribution**	**Age** **range** **[year]** **(mean) years**	**Population**	**Study** **(year)**
			**Lat**	**Mid**	**Med**					
		10.1		5.9		Intraoperative observations	40 F	14-51 (28)	White patients	Daniel (2014)^20^
	0.5	6.4(domal notch) 11. 1(turning point)				Cadaver dissection	13M 7 F	57-88 (74)	White cadavers	Daniel (2014) ^20^
	0.5	9.5	12.1	6.4	4.3	Histologic evaluation	13M 8F	47-86 (66.9)	formaldehyde-fixed Korean cadavers	Kim (2010)^14^
	Males: Right: 0.93 Left :0.98 Female: Right:0.86 Left:0.90	Males: Right: 10.03Left: 9.44Female: Right9.87 Left 9.67	7.14(M) 6.97(F)	4.7	2.89(M) 2.57(F)	Intraoperative measurements	21M 14F	>18	Indian patients	Kasliwal(2018)^22^
4.3						Ultrasound imaging (Tip)	16M 31F	18-70 (35)	Brezilian Patients	Naves (2011)^9^
1.1 1.2						Ultrasound imaging (Tip-supratip)	12M 63F	19-61 (33)	Patients (California)	Kosins^11^ (2016)
0.32						Ultrasound imaging (lateral aspect of lower lateral domes)	6M 12F	16-41 (22)	Caucasian patients	Tasman^8^ (2000)
2.9						CT scan (Tip, skin thickness)	55M 22F	14-68 (28)	Korean patients	Cho7(2011)
	Mean: 1.0 M: 0.9 F: 0.8	Mean: 10.8 M: 11.23 F:10.21	14(M) 12(F)7(M)6(M)	7(M) 6(F)	6(M) 5(F)	Cadaver dissection	22M 14F	20-75 (42)	Fresh Iranian Cadavers	Farahvash21 (2012)
3.04 2.46						Ultrasound imaging (Tip, supratip, skin thickness)	12M 68F	25.1	Iranian patients	Nemati12(2013)
	0.9	10.8(M) 9.7(F)	6.9(M) 6.3(F)	4.6(M) 4.4(F)	3.2(M) 2.9(F)	Cadaver dissection	14F 31M	Not mentioned	Formalin fixed black cadavers	Mc Intosh23(2016)
2.56 2.37	0.51	11.44(M) 10.41(F)	10.89(F)11.9(M)	6.93(F) 7.9(M)	4.38(F) 5.38(M)	Intraoperative measurements. Ultrasound imaging	41F 25M	18-38 (27.82)	Iranian patients	Present study

LC-nostril: the distance from the lower border of lateral crus to the alar rim, Med: medial, mid: middle, Lat: lateral, LCW: lateral crural width, LCT: lateral crural thickness, STT: skin/soft tissue thickness

## Conclusion

The results of the present study revealed some differences in the measured anatomical parameters between Iranians and other ethnic groups (Caucasians and other so-called non-Caucasians). However, drawing a definite conclusion about these variations needs comparative studies with similar samples (cadaver vs. patients) and measurement techniques. It seems that there still exists a scarcity of data and details about this issue.
